# Radiotherapy-Associated Systemic Antitumor Responses Beyond the Classical Abscopal Paradigm

**DOI:** 10.3390/cancers18132098

**Published:** 2026-06-28

**Authors:** Yosuke Dotsu, Kazumasa Akagi, Noritaka Honda, Midori Matsuo, Hirokazu Taniguchi, Shinnosuke Takemoto, Hiroshi Mukae

**Affiliations:** 1 Department of Respiratory Medicine, Nagasaki University Graduate School of Biomedical Sciences, Nagasaki 852-8501, Japan; kaakagi@nagasaki-u.ac.jp (K.A.); nhonda@nagasaki-u.ac.jp (N.H.); mi-shimada@nagasaki-u.ac.jp (M.M.); hirokazu_pc@nagasaki-u.ac.jp (H.T.); shinnosuke-takemoto@nagasaki-u.ac.jp (S.T.); 2 Clinical Oncology Center, Nagasaki University Hospital, Nagasaki 852-8501, Japan; 3 Clinical Research Center, Nagasaki University Hospital, Nagasaki 852-8501, Japan; 4 Department of Respiratory Medicine, Nagasaki Harbor Medical Center, Nagasaki 850-8555, Japan; hmukae@nagasaki-u.ac.jp

**Keywords:** radiotherapy, abscopal effect, systemic antitumor response, immune modulation, non-small cell lung cancer

## Abstract

Radiotherapy (RT) has traditionally been considered a local treatment modality; however, accumulating evidence suggests that it can induce systemic antitumor responses outside the irradiated field. The classical abscopal effect, defined as the regression of non-irradiated tumor lesions following localized RT, has gained renewed attention in the era of immune checkpoint inhibitors (ICIs). Preclinical and clinical studies have demonstrated that RT can promote immunogenic cell death, antigen presentation, cytokine release, and adaptive immune activation, processes that may contribute to systemic antitumor immunity, particularly when combined with ICIs. Clinically apparent abscopal responses remain rare and heterogeneous, and the biological mechanisms underlying these phenomena are not completely understood. Systemic tumor regression has occasionally been observed in clinical settings that do not involve checkpoint blockade, including RT alone, chemotherapy-containing regimens, and central nervous system-directed therapies. These findings suggest that radiation-associated systemic antitumor responses may arise through overlapping immunologic mechanisms involving both innate and adaptive immunity, tumor microenvironment remodeling, and treatment-associated cellular stress. This review summarizes the current clinical evidence and biological mechanisms underlying radiation-associated systemic antitumor responses and discusses emerging concepts extending beyond the conventional RT–immunotherapy paradigm. We further examined unresolved challenges, including treatment heterogeneity, biomarker limitations, pseudoprogression, and difficulties in mechanistic confirmation. Finally, we propose that the broader “beyond-abscopal” framework may serve as a hypothesis-generating conceptual model for investigating systemic tumor responses across diverse treatment contexts while emphasizing the need for cautious interpretation and prospective translational validation.

## Simple Summary

Radiotherapy (RT) has traditionally been regarded as a local treatment modality; however, accumulating evidence suggests that it can induce systemic antitumor responses outside the irradiated field. The abscopal effect, historically considered a rare phenomenon, has attracted renewed attention in the era of immune checkpoint inhibitor (ICI) therapy. Systemic tumor regression has occasionally been observed in treatment settings that do not involve ICIs, including RT alone, chemotherapy-containing regimens, and central nervous system-directed therapies. This review summarizes the current clinical and biological evidence regarding radiation-associated systemic antitumor responses and discusses the mechanisms involving immunogenic cell death, cytokine signaling, tumor microenvironment remodeling, and systemic immune modulation. We also address the major limitations of this review, including the rarity, heterogeneity, and difficulty of mechanistic confirmation of these phenomena. Finally, we propose a broader perspective extending beyond the classical abscopal paradigm, emphasizing the need for cautious interpretation and prospective translational validation.

## 1. Introduction

Traditionally, radiotherapy (RT) has been considered a local treatment modality; however, mounting evidence suggests that it can exert systemic antitumor effects by modulating the immune system [1,2]. Ionizing radiation induces immunogenic cell death (ICD) [3,4], leading to the release of tumor-associated antigens and damage-associated molecular patterns (DAMPs), which promote dendritic cell (DC) maturation and cytotoxic T-cell activation [5,6]. Through these processes, RT has been increasingly recognized as a potential modulator of systemic antitumor immunity beyond the irradiated field [7].

One of the most intriguing clinical manifestations of this phenomenon is the abscopal effect, defined as the regression of nonirradiated tumor lesions following localized RT [8,9]. Although the abscopal effect has been reported in various tumor types, it remains a rare and unpredictable event in clinical practice [10]. In recent years, the integration of immune checkpoint inhibitors (ICIs) with RT has revitalized interest in this phenomenon [11,12], with accumulating reports suggesting enhanced systemic responses in selected patients. These observations have led to the prevailing view that the effective induction of systemic antitumor immunity by RT depends largely on concurrent immune modulation, primarily through ICI-based strategies [13]. Despite these advances, several clinical and biological uncertainties remain. First, systemic tumor responses outside irradiated fields have occasionally been observed even in the absence of immunotherapy, suggesting that RT-induced immune activation may not rely exclusively on ICIs [9,10]. Second, the biological mechanisms underlying these responses are not yet fully understood [1,13], particularly in clinical contexts that fall outside conventional RT-immunotherapy paradigms [7,13]. Third, emerging clinical settings—such as central nervous system (CNS)-directed therapy, chemotherapy-associated immunological effects [4], and tumor–host interactions beyond the primary irradiation site—raise the possibility that systemic antitumor responses likely arise through overlapping immunological, inflammatory, and treatment-associated mechanisms that are more heterogeneous than previously thought.

These uncertainties present significant clinical and conceptual challenges. Occasional systemic tumor regression has been observed during treatment courses that do not include immunotherapy; however, there is no established model for interpreting these findings. In this context, we recently reported the regression of a nonirradiated lung adenocarcinoma during chemoradiotherapy (CRT) for glioblastoma, in the absence of any specific treatment for the lung cancer [14]. Although causality cannot be established from a single case, this observation raises the possibility that radiation-associated systemic antitumor responses may occur outside conventional RT-immunotherapy paradigms.

Building on these observations, this review aims to synthesize the current clinical evidence, summarize the underlying biological mechanisms, and discuss systemic antitumor responses occurring across diverse radiation-associated treatment settings. Importantly, the term “beyond-abscopal” is not intended to define a novel biological entity or distinct mechanistic pathway. Rather, it is proposed as a clinical and conceptual framework for interpreting radiation-associated systemic antitumor responses that occur not only in conventional RT–immune checkpoint inhibitor (ICI) settings but also in contexts such as RT alone, chemotherapy-containing regimens, and central nervous system (CNS)-directed therapies. Although many of the underlying biological processes have been reviewed previously, this perspective aims to integrate these diverse clinical observations and generate testable hypotheses for biomarker development and prospective studies.

## 2. Clinical Evidence of Systemic Antitumor Responses

### Classical Abscopal Effect

The abscopal effect is classically defined as the regression of tumor lesions outside the irradiated field following localized RT, a concept first described by Mole in 1953 [8]. Since its original description, this phenomenon has been documented in various malignancies, including melanoma, lymphoma, and non-small cell lung cancer (NSCLC), primarily as individual case reports and small case series [9,10]. Recently, patient-level analyses and contemporary reviews have further characterized the clinical features, frequency, and limitations of reported abscopal responses, highlighting their rarity, heterogeneity, and challenges associated with establishing causality [15,16]. Notably, only a limited number of well-documented clinical cases have been reported over several decades, underscoring both the rarity of the phenomenon and the restricted evidence base available for mechanistic interpretation [15].

However, the abscopal effect has historically been considered a rare and unpredictable clinical event. Comprehensive reviews have consistently emphasized its low incidence and limited reproducibility in routine clinical practice, particularly in the absence of systemic immune modulation [9,10]. Furthermore, no consistent association has been established between the occurrence of abscopal responses and specific clinical variables—such as tumor histology, radiation dose, fractionation schedules, or treatment timing—underscoring the stochastic nature of this phenomenon [9].

Early clinical observations suggested that abscopal responses could occur following RT alone, including the regression of metastatic lesions after irradiating a primary or distant tumor site [9,10]. These responses, however, remained sporadic, and their clinical significance has been difficult to define due to a lack of prospective validation and the potential contribution of confounding factors, such as spontaneous regression or delayed effects of systemic therapy.

From a mechanistic perspective, the abscopal effect is now believed to be mediated, at least in part, by radiation-induced immune system activation. Ionizing radiation can promote tumor antigen release and enhance antigen presentation, leading to the activation of cytotoxic T-cell-mediated immune responses capable of targeting tumor cells at distant sites [17]. In the absence of additional immune stimulation, these responses are generally insufficient to induce consistent systemic tumor control, which may explain the rarity of clinically significant abscopal effects observed in routine clinical practice.

This low frequency, unpredictability, and limited reproducibility highlight the intrinsic limitations of RT alone in reliably achieving systemic, immune-mediated tumor regression. To provide historical context, Table 1 summarizes major milestones in the development of the abscopal-effect concept and radioimmunotherapy.

## 3. Biological Mechanisms

**Figure 1:**
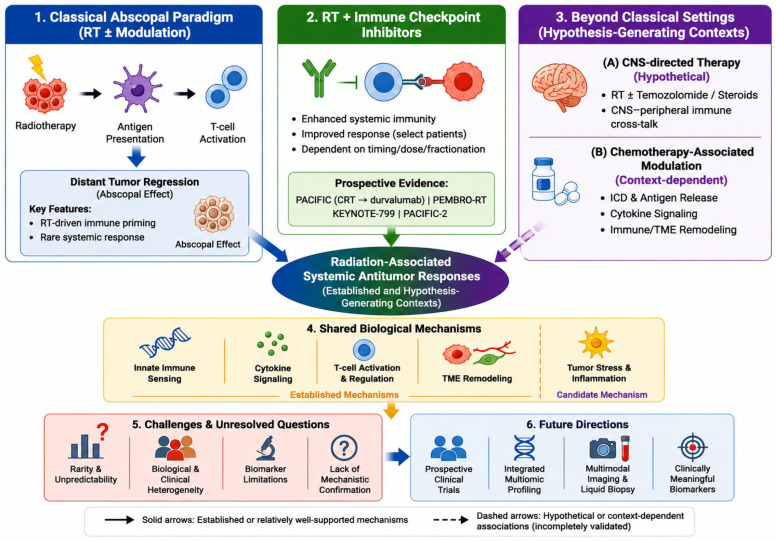
Conceptual framework of radiation-associated systemic antitumor responses beyond the classical abscopal paradigm. The figure summarizes both established and hypothetical mechanisms that may contribute to radiation-associated systemic antitumor responses. Solid arrows indicate established or relatively well-supported pathways, including immunogenic cell death, antigen presentation, T-cell activation, cytokine signaling, cGAS-STING activation, and tumor microenvironment remodeling. Dashed arrows indicate more speculative or context-dependent associations, particularly systemic responses observed in selected non-ICI clinical contexts, including CNS-directed therapy and chemotherapy-containing regimens. This framework is intended as a conceptual model rather than evidence of a unified biological mechanism. Abbreviations: RT, radiotherapy; ICI, immune checkpoint inhibitor; CNS, central nervous system.

Beyond direct cytotoxicity, RT influences tumor immunity, stromal interactions, and inflammatory signaling through several interconnected mechanisms, many of which are well supported in preclinical models but remain incompletely validated as direct causes of clinical systemic tumor regression [17,18]. These mechanisms operate across multiple biological levels, including ICD, innate and adaptive immune activation, microenvironmental remodeling, and systemic inflammatory signaling [1,13]. These established and hypothetical mechanisms are summarized conceptually in Figure 1.

### 3.1. Immunogenic Cell Death

One of the most fundamental mechanisms linking RT to systemic antitumor immunity is the induction of ICD. Unlike conventional apoptotic or necrotic cell death, ICD is characterized by the release of DAMPs, including high-mobility group box 1 (HMGB1) and adenosine triphosphate (ATP), as well as the surface exposure of calreticulin [5,19,20]. These molecules can act as endogenous danger signals that alert and activate the host immune system.

HMGB1 interacts with pattern recognition receptors, such as Toll-like receptor 4 (TLR4), promoting DC maturation and antigen processing [5]. Extracellular ATP acts as a chemoattractant and activation signal for antigen-presenting cells via purinergic receptors [4], while calreticulin exposure on dying tumor cells facilitates phagocytosis by DCs [19]. Together, these signals can establish a proimmunogenic environment that promotes the initiation of an antitumor immune response.

ICD is not exclusive to RT and can also be induced by certain chemotherapeutic agents, suggesting that systemic antitumor effects may arise from mechanisms shared across different treatment modalities [4,5,20]. The extent and quality of ICD induction vary depending on radiation dose, fractionation, and tumor type, contributing to the heterogeneity of downstream immune responses [18].

### 3.2. Antigen Presentation and T Cell Activation

Following ICD, tumor-associated antigens are processed and presented by DCs, leading to the activation of tumor-specific T cells. This process represents a crucial link between local tumor irradiation and systemic immune responses. Preclinical models have shown that local RT activates tumor-associated DCs and supports tumor-specific effector CD8^+^ T-cell responses, indicating that DC-mediated antigen presentation can be critical for radiation-induced antitumor immunity [21,22].

Activated DCs migrate to the draining lymph nodes, where they present tumor-associated antigens to naive T cells, promoting the expansion of cytotoxic CD8^+^ T cells capable of targeting tumor cells at distant sites [23]. RT can further enhance this process by increasing antigen visibility and major histocompatibility complex (MHC) class I expression, thereby making tumors more susceptible to T-cell-mediated antitumor immunity [24].

A landmark experimental study demonstrated that ionizing radiation modulates the tumor peptide repertoire, increases MHC class I expression, and enhances susceptibility to T-cell-mediated antitumor immunity [24]. Importantly, RT has also been associated with increased tumor-infiltrating T cells and immune-mediated tumor control in preclinical settings, supporting its role as a local treatment that can amplify systemic adaptive immune responses. However, in the absence of sufficient costimulatory signals, T-cell activation may be incomplete or transient. This limitation provides a biological rationale for combining RT with immunomodulatory strategies, including ICIs, costimulatory agonists, or agents targeting suppressive and regulatory myeloid cell populations [22,25].

### 3.3. Tumor Microenvironment Modulation

RT exerts profound effects on the TME, influencing immune cell composition, as well as vascular and stromal dynamics. These changes can either facilitate or hinder systemic antitumor responses, depending on the context [26,27].

One key aspect is the modulation of tumor vasculature. RT has been shown to increase vascular permeability and, under certain conditions, promote the normalization of abnormal tumor blood vessels, thereby enhancing immune cell trafficking to tumor sites. Improved perfusion can also facilitate the delivery of systemic therapies and increase oxygenation, further impacting treatment response [28,29].

Furthermore, RT alters the balance of immune cell populations within the TME. RT has been reported to increase effector T-cell infiltration while simultaneously modulating immunosuppressive populations, such as regulatory T cells (Tregs), myeloid-derived suppressor cells, and tumor-associated macrophages. The net biological impact of these changes likely varies depending on the TME and host immune status, which can determine whether the TME becomes immunostimulatory or immunosuppressive [30]. RT also induces the remodeling of the extracellular matrix and stromal compartments, which can influence tumor cell survival, invasion, and metastatic potential. Recent evidence suggests that radiation-induced stromal remodeling may also contribute to resistance against radioimmunotherapy. A recent study identified a population of SFRP2-high cancer-associated fibroblasts (CAFs) induced through PAI-1-dependent signaling after irradiation [31]. These CAFs promoted an immunosuppressive microenvironment and were associated with reduced responsiveness to radioimmunotherapy. These findings suggest that stromal heterogeneity may represent an additional determinant of variability in radiation-associated systemic antitumor responses.

### 3.4. Cytokine and Inflammatory Signaling

Beyond local immune activation, RT can induce inflammatory and cytokine responses that can influence tumor behavior at distant sites. Irradiated tumors release a variety of soluble mediators, including interleukin-1 (IL-1), interleukin-6 (IL-6), tumor necrosis factor-α (TNF-α), and type I interferons, which collectively contribute to systemic immune modulation [17,32].

Among these mediators, type I interferons play a central role in bridging innate and adaptive immunity. The activation of cytosolic DNA-sensing pathways following radiation leads to the production of type I interferons, which promote DC activation and enhance the cross-priming of tumor-specific T cells [18,33]. This pathway has been shown to be essential for generating effective antitumor immune responses following RT in preclinical models. However, the immunological consequences of cGAS-STING activation are not uniformly beneficial. Recent evidence suggests that cGAS-STING-mediated recruitment of γδ T cells may promote radioresistance under specific conditions, underscoring the context-dependent nature of radiation-induced innate immune responses [34].

In parallel, proinflammatory cytokines such as IL-1, IL-6, and TNF-α can modulate immune cell trafficking, alter vascular permeability, and remodel tumor–host interactions at both local and systemic levels. These cytokine-driven effects are also associated with changes in tumor growth dynamics beyond the irradiated field, even in the absence of robust adaptive immune activation [35,36].

Cytokine signaling also has context-dependent effects. While acute inflammatory responses can enhance antitumor immunity, excessive or chronic inflammation can promote tumor progression, immunosuppression, and treatment resistance. In particular, IL-6-mediated signaling has been implicated in tumor proliferation, survival, and immune evasion across various cancer types [37,38].

## 4. Synergy Between Radiotherapy and Immunotherapy

Combining RT with ICIs has become a major focus in modern thoracic oncology and radioimmunotherapy research. This approach aims to convert immunologically “cold” tumors into “hot” tumors, thereby improving responsiveness to immunotherapy [13,17,32]. ICIs targeting programmed cell death protein 1 (PD-1), programmed death-ligand 1 (PD-L1), and cytotoxic T-lymphocyte-associated antigen 4 (CTLA-4) have been extensively studied in this context [39].

### 4.1. Immune Checkpoint Blockade as a Synergistic Partner

RT-induced immune activation is frequently counterbalanced by inhibitory pathways within the TME, including the upregulation of PD-L1 on both tumor and immune cells. This provides a biological rationale for combining RT with ICIs [25,40].

In addition to PD-1/PD-L1 signaling, CTLA-4 plays an essential role in regulating early T-cell activation. CTLA-4 blockade has been shown to enhance T-cell priming and expansion, particularly in the context of increased antigen availability following RT. Early experimental studies demonstrated that RT combined with immune modulation increases systemic tumor control via T-cell-dependent mechanisms, providing a mechanistic basis for radiation-induced systemic immunity [41].

Subsequent work established that dual ICIs combined with RT can activate complementary immune pathways, resulting in improved tumor control compared to either modality alone [42]. More recently, translational and clinical studies have supported the relevance of these mechanisms in human tumors, suggesting that RT-induced immune priming can enhance responsiveness to ICIs in selected patient populations [1,7].

### 4.2. Timing, Dose, Fractionation

Treatment timing, dose, fractionation, and irradiated volume appear to influence the efficacy of RT-ICI combinations. While preclinical and clinical studies support the immunomodulatory potential of RT, a universally optimal regimen has yet to be established. Recent consensus discussions have emphasized that radiation should be viewed as an immunomodulatory “drug” whose dose and fractionation likely need to be tailored to the specific clinical context, rather than applied uniformly across all clinical settings [43].

The optimal sequencing of RT and ICIs remains an active area of investigation. Preclinical data suggest that concurrent or closely sequenced treatment may maximize immune activation by aligning radiation-induced antigen release and innate immune stimulation with ICIs. Clinically, the strongest evidence comes from unresectable stage III NSCLC, where the PACIFIC trial established consolidation durvalumab following definitive CRT as the standard of care. In the PACIFIC trial, durvalumab was initiated 1 to 42 days after CRT; progression-free survival and overall survival improved significantly compared to placebo, and 5-year follow-up confirmed this durable benefit [44,45,46].

However, the PACIFIC trial should be interpreted as evidence supporting sequential consolidation immunotherapy after CRT, rather than proof that immediate or concurrent administration is universally optimal. Exploratory and real-world analyses have raised the possibility that early initiation may be associated with favorable outcomes; however, these findings remain susceptible to selection bias and do not definitively establish an optimal starting time [47]. Concurrent strategies are also being explored. The KEYNOTE-799 trial evaluated pembrolizumab administered with concurrent CRT in unresectable stage III NSCLC, demonstrating promising antitumor activity and manageable safety in a nonrandomized phase II setting; however, randomized validation is still needed [48].

Radiation dose and fractionation critically influence the immunological consequences of RT. Preclinical studies have shown that fractionated regimens may be more effective than single-dose RT at inducing immune-mediated abscopal responses when combined with CTLA-4 blockade. Dewan et al. demonstrated that fractionated RT, but not single-dose RT, induced an immune-mediated abscopal effect when combined with an anti-CTLA-4 antibody, supporting the concept that fractionation can shape systemic immune responses [49].

Mechanistically, the radiation dose per fraction may determine whether RT promotes or suppresses antitumor immunity. Vanpouille-Box et al. demonstrated that high single doses of radiation above approximately 12–18 Gy induce the DNA exonuclease TREX1, which degrades cytosolic DNA and attenuates cGAS-STING-dependent type I interferon signaling. This finding provides a mechanistic rationale for why intermediate hypofractionated regimens may be more immunogenic than excessively high single-dose regimens, although direct clinical validation remains incomplete [18].

Current evidence supports the importance of timing, dose, and fractionation in RT-ICI synergy; however, the optimal strategy likely varies depending on the disease setting, tumor burden, immune context, and therapeutic intent.

### 4.3. Clinical Evidence

Evidence supporting RT-ICI synergy has accumulated across various disease settings, particularly in NSCLC. The PEMBRO-RT trial evaluated pembrolizumab with or without stereotactic body radiotherapy (SBRT) to a single tumor site prior to immunotherapy in metastatic NSCLC. Although the primary endpoint was not met in the intention-to-treat population, the trial reported a clinically meaningful improvement in response among patients who received SBRT, supporting the hypothesis that RT can enhance systemic immune responsiveness in selected patients [50].

This signal was reinforced by a pooled analysis of the PEMBRO-RT and MD Anderson randomized phase II trials, which reported improved response and survival outcomes when RT was added to pembrolizumab in patients with metastatic NSCLC. The authors noted the limited sample size and the need for validation in larger randomized trials, emphasizing that while RT-ICI synergy in metastatic disease remains promising, it is not yet definitively established [51]. In unresectable stage III NSCLC, the PACIFIC trial provides the strongest clinical evidence for integrating RT and immunotherapy. Consolidation durvalumab following concurrent platinum-based CRT significantly improved progression-free survival and overall survival compared to placebo. The 5-year update confirmed a durable survival benefit, establishing this approach as a global standard of care [46,48]. Real-world evidence has further supported the generalizability of this strategy, with mature data from PACIFIC-R showing outcomes broadly consistent with the PACIFIC trial [52].

Meanwhile, more intensive approaches incorporating ICIs concurrently with CRT have yielded more nuanced results. KEYNOTE-799, a nonrandomized phase II trial, demonstrated promising antitumor activity and a manageable safety profile for pembrolizumab plus concurrent CRT in unresectable stage III NSCLC; however, the lack of a randomized comparator limits definitive interpretation [50]. In contrast, the phase III PACIFIC-2 trial, which evaluated concurrent durvalumab with platinum-based CRT followed by consolidation durvalumab, failed to clearly demonstrate superiority over the standard sequential consolidation strategy. This underscores that earlier or concurrent ICI administration is not inherently superior [53].

Collectively, current clinical evidence supports RT-ICI integration as a major therapeutic advance, particularly in unresectable stage III NSCLC. However, the data also reveal substantial heterogeneity across disease stages, radiation strategies, ICI timing, and patient selection. Consequently, the clinical impact of RT-ICI combinations varies depending on the disease setting, radiation strategy, and host immune characteristics, highlighting the need for ongoing optimization of treatment sequencing, radiation parameters, biomarkers, and patient selection. Although much of the available evidence has been generated in NSCLC, recent phase III studies in other tumor types have further expanded the clinical relevance of RT–ICI integration. In locally advanced cervical cancer, the phase III KEYNOTE-A18 trial demonstrated significant improvements in progression-free and overall survival with pembrolizumab combined with chemoradiotherapy followed by pembrolizumab maintenance [54]. Similarly, the phase III NIVOPOST-OP trial reported improved disease-free survival with the addition of nivolumab to postoperative chemoradiotherapy in high-risk resected head and neck squamous cell carcinoma [55]. These findings suggest that the immunomodulatory interactions between RT and ICIs may extend beyond NSCLC and support the broader clinical applicability of radioimmunotherapy strategies across multiple tumor types. However, the interpretation of these studies requires caution because substantial heterogeneity exists in patient populations, radiation dose and fractionation schedules, treatment sequencing, and response assessment methods. Furthermore, many studies were not specifically designed to evaluate radiation-associated systemic antitumor responses, limiting definitive conclusions regarding incidence, causality, and underlying mechanisms.

### 4.4. Limitations and Unresolved Questions

Despite encouraging clinical and translational data, several unresolved questions continue to limit the broader application of RT-ICI combinations. First, the benefits are not universal. Even in unresectable stage III NSCLC, where consolidation durvalumab following CRT is the established standard of care, a substantial proportion of patients relapse, and durable disease control is achieved in only a subset of patients. The 5-year update of the PACIFIC trial confirmed long-term benefits but also showed that many patients still experience relapse or die, underscoring the need for better patient selection and additional therapeutic strategies [46].

Second, the optimal timing of ICI administration relative to RT remains unclear. Although early or concurrent administration is biologically appealing, recent phase III data have challenged the assumption that “earlier is necessarily better.” The PACIFIC-2 trial showed that initiating durvalumab concurrently with CRT provided no additional benefit over CRT followed by consolidation durvalumab, supporting the current PACIFIC-style sequential approach as the standard strategy [53]. Similarly, the CheckMate 73L trial failed to demonstrate the superiority of concurrent nivolumab-based CRT followed by nivolumab (with or without ipilimumab) compared to CRT followed by durvalumab, further highlighting the complexity of optimizing treatment sequencing [56,57].

Third, the optimal radiation dose, fractionation schedule, target lesion selection, and irradiated volume remain poorly defined. Preclinical data suggest that radiation parameters strongly influence immune activation, primarily through cytosolic DNA sensing and type I interferon signaling. Translating these principles to the clinic remains a challenge. Therefore, RT should not be viewed as a uniform immune stimulus; rather, its systemic immunological consequences likely depend on dose, fractionation, tumor burden, anatomical site, and the preexisting immune context.

Fourth, predictive biomarkers remain inadequate. PD-L1 expression, tumor mutational burden (TMB), circulating tumor DNA (ctDNA) dynamics, lymphocyte counts, and T-cell receptor (TCR) clonality have all been explored, but none has yet provided a universally reliable method for selecting patients most likely to benefit from RT-ICI combinations. Furthermore, the emergence of targeted consolidation approaches—such as osimertinib following CRT in unresectable, EGFR-mutated stage III NSCLC—further complicates the role of immunotherapy in molecularly defined subgroups and underscores the need to integrate genomic stratification into future RT-systemic therapy trials [58].

Finally, the RT-ICI paradigm does not fully account for all systemic antitumor responses observed in clinical practice. As discussed above, systemic tumor regression can occur in settings without ICIs, suggesting that cytokine-mediated signaling, TME remodeling, innate immune activation, and intrinsic tumor dynamics may all contribute to abscopal or “beyond-abscopal” responses. Therefore, future studies must move beyond simply asking whether RT and ICIs are synergistic; instead, they should define which patients, which tumors, which radiation parameters, and which systemic partners are required to convert local therapy into durable systemic tumor control.

## 5. Beyond the Abscopal Paradigm

Although RT–ICI combinations represent the most extensively investigated clinical setting for radiation-associated systemic antitumor responses, systemic tumor regression has occasionally been observed outside conventional RT–ICI paradigms. The beyond-abscopal concept has been proposed as a practical framework for interpreting radiation-associated systemic responses observed in different clinical settings. Rather than representing a distinct biological entity, this framework aims to facilitate the interpretation of observations that fall outside the conventional RT–ICI paradigm, while acknowledging substantial mechanistic uncertainty. To clarify the conceptual distinction among the classical abscopal effect, RT–ICI-associated systemic responses, non-ICI systemic responses, and the proposed beyond-abscopal framework, a comparative overview is presented in Table 2.

Previous reviews by Siva, Reynders, and Demaria collectively established the concepts of clinical rarity, limited reproducibility, and immune-mediated mechanisms underlying the abscopal effect, providing a foundation for the present framework [1,2,9,10].

Therefore, the present framework should be viewed as an extension of earlier abscopal and radioimmunotherapy concepts rather than a replacement.

The classic abscopal model may not fully capture the spectrum of systemic tumor responses observed in current clinical practice. Although this phenomenon has been increasingly associated with immune-mediated mechanisms, particularly in the era of ICIs, its clinical occurrence remains relatively rare and unpredictable.

Growing evidence also suggests that systemic tumor responses can occur in clinical settings not readily explained by canonical immune checkpoint-mediated mechanisms. For example, tumor regression outside irradiated fields has been reported following RT alone or combined with nonimmunotherapeutic modalities, including cytotoxic chemotherapy, indicating that these effects are not solely dependent on ICIs [5,9,10]. Furthermore, emerging clinical observations in unconventional settings, such as CNS-directed therapy, raise the possibility that systemic tumor responses may arise through the interplay of RT-associated immune and inflammatory responses, treatment-related cellular stress, and tumor–host interactions.

These observations suggest that systemic tumor responses may involve treatment- and host-related mechanisms [17,32]. From this perspective, the classic abscopal effect can be viewed as one manifestation of a broader category of treatment-associated systemic tumor responses.

In this section, we expand the current paradigm beyond the classic abscopal model by examining four main domains: (i) systemic responses occurring in the absence of ICIs, (ii) immune modulation associated with CNS-directed therapies, (iii) chemotherapy-induced immune effects, and (iv) the distinction between true systemic tumor regression and pseudo-responses or clinical confounding factors.

### 5.1. Non-ICI Systemic Responses

Historically, abscopal responses were reported long before the advent of ICIs, although they were rare, unpredictable, and primarily documented in individual case reports or small case series. Systematic and clinical reviews have emphasized that the regression of nonirradiated tumor lesions can occur following localized RT alone; however, its incidence is low, and its reproducibility remains limited [9,10,15,16]. These observations are important because they indicate that ICIs are not an absolute prerequisite for systemic tumor regression.

The rarity of these responses suggests that RT alone is generally insufficient to induce durable systemic tumor control. Experimental evidence supports the view that abscopal responses are at least partially immune-mediated; in a preclinical model, distant tumor inhibition following localized irradiation required T cells, providing a mechanistic basis for radiation-induced systemic immunity even in the absence of ICIs [41]. However, in clinical settings, this immune activation may not consistently overcome tumor immune escape, suppressive TMEs, or insufficient antigenicity.

Therefore, non-ICI-related systemic responses should not be interpreted merely as exceptions to the ICI-driven abscopal model. Instead, they likely represent a manifestation of radiation-induced systemic effects, which range from classic immune-mediated abscopal responses to more complex interactions among RT, host immunity, inflammatory signaling, and tumor biology. However, caution is warranted when interpreting these observations. Much of the evidence supporting RT-alone-associated systemic responses derives from individual case reports and small retrospective studies. Consequently, the true incidence, reproducibility, and predictors of these responses remain unknown.

### 5.2. CNS-Directed Therapy and Systemic Immunologic Alterations

The potential relationship between CNS-directed therapy and extracranial systemic antitumor responses remains highly speculative. Although the CNS has historically been considered an immune-privileged site, recent studies have demonstrated active immune surveillance and communication between the CNS, meningeal lymphatic vessels, and peripheral immune compartments [59,60]. These findings provide a biological rationale for considering whether CNS-directed treatments might be associated with systemic immunologic alterations; however, they do not establish that CNS irradiation induces reproducible extracranial antitumor responses.

In patients with glioblastoma, standard treatment with RT and temozolomide (TMZ) can alter systemic immunity, most notably through treatment-related lymphopenia and changes in circulating immune cell populations [61,62,63,64]. These systemic immunological changes may influence host–tumor interactions, but their relationship to extracranial tumor regression remains uncertain and has not been prospectively validated.

In this context, our recent case provides a clinically relevant example of systemic tumor regression occurring outside the conventional RT-ICI framework. We observed the regression of a nonirradiated lung adenocarcinoma during glioblastoma-directed CRT with TMZ, without any specific treatment for the lung cancer [14]. Although this temporal association raises the possibility of treatment-induced systemic immunological alterations, causality cannot be established from a single case, and alternative explanations cannot be completely excluded. Therefore, this observation should not be regarded as evidence of a distinct CNS-directed or beyond-abscopal phenomenon.

Overall, CNS-directed therapy should be considered in a speculative and exploratory context within the broader framework of radiation-associated systemic antitumor responses. Further prospective studies incorporating longitudinal imaging, immune monitoring, and molecular biomarkers are required to determine whether such observations represent reproducible biological events or rare coincidental clinical findings. This caution is warranted because the current evidence base remains limited and largely hypothesis-generating. Most observations originate from isolated case reports or small clinical series, and causal relationships between CNS-directed therapy and extracranial tumor regression cannot be established.

### 5.3. Chemotherapy-Induced Immune Effects

Certain cytotoxic agents exert immunomodulatory effects that extend beyond direct tumor cell killing. They can also modulate antitumor immunity through mechanisms overlapping with those induced by RT, including ICD and shifts in immune cell composition [4,5,21,22].

In addition to promoting tumor antigen release, chemotherapy can deplete immunosuppressive cell populations, alter tumor antigenicity, and modify systemic immune homeostasis [4,65]. These observations support the biological plausibility of systemic tumor responses in treatment settings that do not involve ICIs.

The immunological effects of TMZ are particularly complex and context-dependent. TMZ can induce lymphodepletion and alter immune cell dynamics, potentially enhancing antitumor immunity in some contexts while impairing immune surveillance in others [63,66]. In glioblastoma, RT combined with TMZ is associated with systemic immunological alterations, including treatment-related lymphopenia and shifts in circulating immune cell populations [61,62,63,64,65,66,67]. The interpretation of these observations is further complicated by the potential interactions between radiation, chemotherapy, and delayed systemic treatment effects, making the attribution of causality inherently difficult in many reported cases.

### 5.4. Systemic Response Versus Pseudo-Response and Confounders

Interpreting “beyond-abscopal” phenomena remains challenging, as apparent systemic tumor regression can stem from various clinical and radiographic confounding factors. The apparent regression of a nonirradiated lesion may reflect measurement variability, differences in imaging techniques, inflammatory changes, necrosis, tumor growth kinetics, delayed effects of prior therapy, spontaneous regression, or lesion misclassification. Therefore, objective response assessment should rely on standardized imaging criteria to minimize variability and bias [68].

In CNS tumors, pseudoprogression following CRT is a well-recognized phenomenon that typically occurs within weeks to months after treatment and can complicate the interpretation of radiographic changes [69,70]. Rather than true tumor progression or regression, these treatment-related imaging changes reflect inflammation, vascular permeability, and therapy-induced tissue damage.

For extracranial lesions, a rigorous assessment must include serial imaging, objective size measurements using standardized criteria, a careful review of the treatment timeline, and the exclusion of direct radiation exposure or scatter dose, where applicable. Additionally, alternative explanations such as infection, inflammation, infarction, hemorrhage, or the effects of prior systemic therapies should be considered. Whenever possible, tissue confirmation and the integration of molecular or immunologic biomarkers can help distinguish true tumor regression from a pseudo-response.

**Figure 2:**
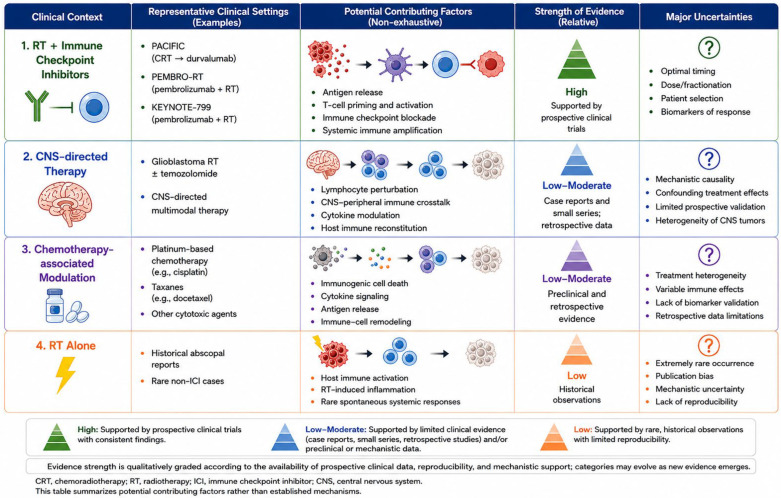
Comparative overview of clinical contexts associated with radiation-associated systemic antitumor responses. This figure provides a conceptual comparison of clinical settings in which radiation-induced systemic antitumor responses have been observed or proposed, including RT–ICI combinations, CNS-directed therapies, chemotherapy-associated treatment contexts, and RT alone. The evidence levels are qualitative categories based on the current availability of prospective clinical data, retrospective or case-based evidence, mechanistic support, and reproducibility. “High” indicates support from prospective clinical trials; “Low–Moderate” indicates limited clinical evidence supported mainly by case reports, small series, retrospective analyses, or preclinical findings; and “Low” indicates rare historical observations with limited reproducibility. The listed contributing factors should be interpreted as plausible mechanisms rather than proven causal pathways. Abbreviations: CRT, chemoradiotherapy; RT, radiotherapy; ICI, immune checkpoint inhibitor; CNS, central nervous system.

Accordingly, careful exclusion of alternative explanations and rigorous clinical assessments are essential when interpreting radiation-associated systemic tumor responses. Figure 2 summarizes the proposed interpretive framework, and practical considerations for clinical evaluation are outlined in Table 3.

## 6. Clinical Translation and Future Perspective

### 6.1. Biomarkers and Clinical Indicators

Reliable biomarkers capable of identifying or mechanistically confirming RT-associated systemic antitumor responses are still lacking. Although the immunological effects of RT have been extensively investigated, most available biomarkers were developed in the context of ICI therapy rather than for radiation-induced systemic responses. Furthermore, radiation can induce both immunostimulatory and immunosuppressive effects, complicating the interpretation of candidate biomarkers across different treatment settings. Therefore, particularly for cases occurring outside the conventional RT-ICI paradigm, clinically meaningful surrogate markers remain insufficiently established [71,72].

Several candidate biomarkers have demonstrated potential utility, including peripheral lymphocyte dynamics, changes in lymphocyte subsets, cytokine- or interferon-related signatures, TMB, PD-L1 expression, ctDNA, and TCR repertoire dynamics. No single biomarker has yet proven reliable across all treatment settings. For example, TMB and PD-L1 expression are established biomarkers for ICIs, but their performance varies depending on the cancer type, assay platform, treatment setting, and tumor immune context. Similarly, ctDNA kinetics and TCR repertoire changes are promising tools for monitoring tumor burden and adaptive immune activation, but their role in confirming radiation-associated systemic immune responses remains to be explored [73,74].

Compounding this issue, the interpretation of candidate biomarkers is further complicated by substantial heterogeneity in tumor biology, radiation dose and fractionation, timing relative to systemic therapy, and host immune status. Previous clinical and translational studies have demonstrated that radiation parameters and treatment sequencing can differentially regulate immune activation, including cGAS-STING signaling and T-cell priming [18,43]. Furthermore, corticosteroid exposure and radiation-induced lymphopenia can significantly influence immune monitoring results and clinical interpretation [72,75]. Therefore, biomarker development in this field will likely require multimodal and longitudinal assessment strategies rather than reliance on a single static parameter.

Among currently available clinical indicators, peripheral lymphocyte dynamics have garnered particular interest as potential surrogate markers for systemic immune activation. RT can exert both immunostimulatory and immunosuppressive effects on circulating immune cells, and radiation-induced lymphopenia is generally associated with poorer clinical outcomes [71]. The transient preservation or alteration of peripheral lymphocyte populations during treatment has occasionally been observed in association with systemic tumor responses, particularly in exploratory studies evaluating RT-immune interactions [43].

However, peripheral blood biomarkers must be interpreted with caution. Changes in lymphocyte counts can be influenced by various confounding factors, including corticosteroid exposure, infection, chemotherapy, bone marrow suppression, and physiological stress responses [75]. Moreover, peripheral immune cell dynamics do not necessarily reflect intratumoral immune activity or antigen-specific T-cell responses within the TME. Therefore, while longitudinal lymphocyte monitoring can provide indirect evidence of systemic immune modulation, it does not necessarily capture intratumoral antigen-specific immune activity.

Cytokines and inflammatory signaling markers have also been investigated as potential surrogate markers. The radiation-induced release of type I interferons and inflammatory cytokines, including IL-1, IL-6, and TNF-α, may reflect the activation of innate immune pathways, such as cGAS-STING signaling [18,33]. These inflammatory mediators can exert varying immunological effects depending on the treatment setting and host factors, and they may also contribute to immunosuppression or tumor-promoting inflammation [39,40].

Emerging approaches, such as liquid biopsy and TCR repertoire analysis, may provide further insights into treatment-associated systemic immune responses. In this context, liquid biopsy refers to minimally invasive blood-based analyses, including circulating tumor DNA (ctDNA), circulating tumor cells, circulating immune cells, soluble cytokines, and extracellular vesicle-derived biomarkers. These approaches may enable the longitudinal assessment of tumor burden, treatment response, immune activation, and disease evolution without repeated tissue sampling. Dynamic changes in ctDNA levels have been linked to treatment response across various malignancies, whereas the expansion of specific TCR clones following RT may reflect antigen spreading and adaptive immune activation [74,76]. However, the prospective validation of these approaches in the context of radiation-associated systemic antitumor responses remains limited. In addition to blood-based biomarkers, multi-omic and spatial immune profiling approaches may help characterize treatment-induced immune activation and resistance mechanisms more comprehensively than single biomarkers [77]. These approaches may also help distinguish systemic immune activation from nonspecific inflammatory or treatment-related changes, although their clinical utility remains investigational and requires further validation.

Advanced imaging biomarkers may also help distinguish true systemic responses from pseudoprogression or treatment-related changes. Standardized assessment frameworks, such as the updated RANO 2.0 criteria, have improved the interpretation of posttreatment imaging findings, particularly in neuro-oncology settings [70].

Given these limitations, biomarker development should prioritize longitudinal and multimodal approaches, particularly ctDNA kinetics and immune signatures. Serial ctDNA monitoring may provide a quantitative measure of systemic tumor burden, molecular response, minimal residual disease, and early relapse, whereas immune signatures, including peripheral immune cell subsets, interferon- or cytokine-related transcriptional programs, TCR repertoire dynamics, and tissue-based immune infiltration patterns, may help characterize treatment-induced immune activation. Integrating ctDNA dynamics, immune signatures, imaging findings, and clinical context may improve the assessment of radiation-associated systemic responses and help distinguish true antitumor activity from treatment-related changes in the future.

In our previously reported case of a nonirradiated lung adenocarcinoma regressing during glioblastoma-targeted CRT, transient changes in peripheral lymphocyte proportions were observed during extracranial tumor regression [14]. While these findings cannot establish causality, they illustrate both the potential utility and the limitations of longitudinal immune monitoring for the exploratory assessment of treatment-associated systemic immunological alterations.

Overall, clinically validated biomarkers capable of reliably identifying radiation-associated systemic antitumor responses are still lacking.

### 6.2. Clinical Implications

The recognition of RT-associated systemic immune effects is increasingly influencing strategies for combining local and systemic therapies. RT can act as an immunomodulatory intervention through mechanisms such as ICD, antigen presentation, cytokine release, and TME remodeling [32,43]. These observations have heightened interest in integrating RT with ICIs and other systemic therapies.

An important clinical consideration is optimizing combination treatment design. Experimental and translational studies suggest that radiation dose, fractionation, target volume, and treatment sequencing likely influence systemic immune activation [23,50]. Specifically, excessively high single doses can induce TREX1 expression and attenuate cGAS-STING-mediated interferon signaling, highlighting the importance of biologically appropriate radiation scheduling [18]. Similarly, concurrent versus sequential RT-ICI strategies may differentially affect efficacy and toxicity, though the optimal approach remains unclear [43].

Clinical experience in thoracic oncology further illustrates the promise and limitations of integrating RT with systemic immunotherapy. Although approaches such as durvalumab consolidation following CRT have improved outcomes in unresectable stage III NSCLC, clinical responses remain substantially heterogeneous [44,45,46]. Overall, radiation-associated systemic immune activation is unlikely to occur uniformly across all patients and may depend on tumor biology, treatment sequencing, and host immune status.

Therefore, patient selection remains a major challenge. Baseline immune status, the tumor immune microenvironment, radiation-induced lymphopenia, prior therapies, and treatment sequencing can all influence systemic immune responses following RT [43,72]. The broader “beyond-abscopal” perspective proposed in this review raises the possibility that clinically significant systemic immune modulation may also occur outside conventional RT-ICI settings, including during CNS-directed therapy or chemotherapy-containing regimens.

These findings support the continued development of integrated treatment strategies combining RT with systemic immune modulation. Currently, however, the available evidence remains largely exploratory. Prospective validation with standardized immune monitoring and biomarker-driven patient selection will be essential before these approaches can be widely incorporated into routine clinical practice.

### 6.3. Limitations and Unresolved Questions

Several key biological and clinical uncertainties continue to limit the interpretation of radiation-associated systemic antitumor responses. First, the precise mechanisms underlying these phenomena are not fully understood. Although preclinical studies have demonstrated the potential roles of ICD, antigen presentation, cytokine signaling, and cGAS-STING pathway activation, direct mechanistic confirmation in human clinical settings remains limited [18,25]. Furthermore, systemic tumor regression likely reflects complex interactions among RT, systemic therapy, host immunity, and tumor biology, rather than a single biologically conserved mechanism.

Another major limitation is the rarity and unpredictability of clinically apparent abscopal or “beyond-abscopal” responses. Most available evidence consists of isolated case reports, retrospective observations, and exploratory translational analyses [9,10]. Collectively, these limitations highlight the need for prospectively designed studies with predefined assessments of non-irradiated lesions, standardized response criteria, and integrated biomarker analyses to improve the quality and interpretability of future evidence. Consequently, the true incidence of radiation-associated systemic antitumor responses remains uncertain.

Publication bias is also a concern. Dramatic or unexpected systemic responses are more likely to be recognized and reported, whereas negative or clinically ambiguous cases may go unpublished. Furthermore, interpreting systemic tumor regression can be complicated by pseudoprogression, spontaneous regression, delayed treatment effects, imaging variability, and other clinical confounding factors [69,70].

The beyond-abscopal framework proposed in this review should be regarded as a hypothesis-generating conceptual model rather than a validated biological category. Further validation will require prospective studies incorporating standardized response assessments, careful exclusion of confounding factors, and integrated biomarker analyses.

Finally, there is significant heterogeneity regarding tumor type, radiation dose and fractionation, treatment sequencing, concurrent systemic therapies, and host immune status. This heterogeneity complicates cross-study comparisons and limits the development of standardized predictive biomarkers or treatment strategies.

### 6.4. Future Directions

It remains unclear whether these responses represent reproducible biological phenomena or rare, context-dependent events. Recent trials on locally advanced NSCLC have illustrated both the promise and the complexity of combining RT with ICIs. KEYNOTE-799 suggested antitumor activity and manageable safety with pembrolizumab plus concurrent CRT, whereas PACIFIC-2 showed no additional benefit when durvalumab was administered from the start of concurrent CRT. This reinforces that the optimal timing and design of these combinations remain unresolved [48,53].

Prospective studies should incorporate predefined evaluations of non-irradiated lesions, standardized imaging criteria, and longitudinal biospecimen collection. Recent translational studies indicate that integrated tissue- and blood-based immune analyses are increasingly feasible in prospective RT-ICI studies. For example, a recent phase II trial using serial tissue and blood biospecimens in metastatic NSCLC showed that SBRT followed by pembrolizumab can be evaluated using integrated multiomic immune profiling, providing a blueprint for future radioimmunotherapy studies [77].

Immune profiling should include peripheral lymphocyte dynamics, immune cell subsets, cytokine- or interferon-related signatures, TCR repertoire analysis, and ctDNA monitoring. TCR repertoire studies support the concept that radiation and ICIs can reshape antitumor T-cell responses, while ctDNA-based minimal residual disease assessment is emerging as a promising tool for monitoring treatment response and relapse risk following CRT and immunotherapy [76,78].

Finally, future translational research must clarify which treatment contexts are most likely to elicit treatment-associated immune and inflammatory responses. This includes not only conventional RT-ICI combinations but also CNS-directed therapy, chemotherapy-containing regimens, and other immunomodulatory approaches. These studies should aim to identify patient subgroups, radiation schedules, and biomarker profiles associated with systemic responses. This framework may guide prospective studies comparing systemic responses across different treatment settings. For example, RT–ICI-associated responses may show stronger adaptive immune activation, TCR clonal expansion, and interferon-related signatures, whereas non-ICI responses may be more strongly associated with inflammatory signaling, immunogenic cell death markers, or treatment-related immune remodeling. Similarly, systemic responses observed after CNS-directed therapy could be tested for associations with peripheral immune alterations, cytokine dynamics, ctDNA clearance, or shared TCR clonotypes preceding extracranial tumor regression. These hypotheses can be evaluated using predefined imaging of irradiated and non-irradiated lesions, serial ctDNA monitoring, paired tissue and blood immune profiling, TCR repertoire analysis, cytokine panels, and spatial immune profiling. Until these data become available, the “beyond-abscopal” concept should currently be viewed as a preliminary interpretive model rather than a basis for routine clinical decision-making.

## 7. Conclusions

RT can occasionally contribute to systemic antitumor responses beyond the irradiated field, even outside conventional RT-ICI settings. Although the underlying biology remains uncertain, these observations suggest that local therapy can influence systemic tumor behavior through broader immune and inflammatory interactions than previously recognized. Further translational and prospective studies are needed to determine when these responses are clinically meaningful and reproducible.

## Figures and Tables

**Table 1:** Historical milestones in abscopal-effect research and radioimmunotherapy development.

Year	Milestone	Conceptual Significance
1953	Mole described the abscopal effect	Introduced the concept of effects occurring outside the irradiated field
2004	Preclinical evidence showed immune-mediated distant tumor inhibition after local RT	Supported an immunological basis for systemic effects
2012	Clinical abscopal response reported with radiotherapy and CTLA-4 blockade in melanoma	Linked RT, checkpoint blockade, and systemic antitumor immunity
2015	Siva et al. and Reynders et al. reviewed clinical evidence and limitations	Emphasized rarity, heterogeneity, and interpretive challenges
2015	Demaria, Golden, and Formenti discussed local RT in cancer immunotherapy	Reframed RT as an immunomodulatory treatment
2017	PACIFIC established consolidation durvalumab after chemoradiotherapy in unresectable stage III NSCLC	Established a major clinical paradigm for RT–ICI integration
2018–2021	Trials such as PEMBRO-RT and pooled analyses evaluated RT plus pembrolizumab in metastatic NSCLC	Provided prospective evidence for systemic immune enhancement in selected patients
2020s	Multiomic, ctDNA, and immune-monitoring approaches emerged	Shifted the field toward mechanistic validation and biomarker-driven investigation

Abbreviations: RT, radiotherapy. ICI, Immune checkpoint inhibitor. CTLA-4, Cytotoxic T-lymphocyte-associated antigen 4. NSCLC, Non-small cell lung cancer. DNA, Deoxyribonucleic acid.

**Table 2:** Comparison of clinical contexts within radiation-associated systemic antitumor responses.

Domain	Classical Abscopal Effect	RT + ICI Systemic Responses	Non-ICI Systemic Responses	Proposed Beyond-Abscopal Framework
Definition	Regression of non-irradiated lesions after localized RT	Systemic responses enhanced by RT–ICI interaction	Systemic responses outside ICI exposure	Operational framework integrating heterogeneous RT-associated systemic responses
Typical clinical setting	RT alone, historical case reports	RT with PD-1/PD-L1 or CTLA-4 blockade	RT alone, RT + chemotherapy, CNS-directed therapy	Cross-context clinical classification
Evidence Level	Low	Moderate to high	Low to moderate	Conceptual/hypothesis-generating
Mechanistic support	Immune-mediated basis supported by preclinical data; limited clinical validation	Strong biological rationale; prospective clinical evidence in selected settings	Plausible but heterogeneous; limited validation	Integrative model; not a distinct mechanism
Key uncertainties	Rarity, reproducibility, confounding	Optimal dose, timing, patient selection, biomarkers	Causality, alternative explanations, reproducibility	Risk of overgeneralization, need for operational criteria and prospective validation
Intended purpose	Describe a rare phenomenon	Therapeutic strategy to enhance systemic immunity	Identify observations outside RT–ICI paradigm	Organize, compare, and guide future investigation

Abbreviations: RT, radiotherapy. ICI, Immune checkpoint inhibitor. CNS, Central nervous system. CTLA-4, Cytotoxic T-lymphocyte-associated antigen 4.

**Table 3:** Proposed considerations for interpreting radiation-associated systemic antitumor responses.

Consideration	Purpose
Anatomical separation	Exclude local RT effect
Objective response assessment	Standardize evaluation
Scatter-dose review	Exclude unintended irradiation
Treatment timeline review	Exclude delayed systemic therapy effects
Longitudinal confirmation	Reduce transient findings
Biomarker support (when available)	Increase biological plausibility

Abbreviations: RT, radiotherapy.

## Data Availability

No new datasets were generated or analyzed.

## Abbreviations

The following abbreviations are used in this manuscript.

ICI	Immune checkpoint inhibitor
RT	Radiotherapy
ICD	Immunogenic cell death
DAMP	Danger-associated molecular pattern
CNS	Central nervous system
NSCLC	Non-small cell lung cancer
cGAS-STING	Cyclic GMP-AMP synthase stimulator of interferon genes
HMGB1	High-mobility group box 1
ATP	Adenosine Triphosphate
TLR-4	Toll-like receptor 4
DC	Dendritic cell
MHC	Major histocompatibility complex
TME	Tumor microenvironment
MDSC	Myeloid-derived suppressor cell
IL-1	Interleukin-1
IL-6	Interleukin-6
TNF-α	Tumor necrosis factor-α
DNA	Deoxyribonucleic acid
PD-1	Programmed death-1
PD-L1	Programmed death-ligand 1
CTLA-4	Cytotoxic T-lymphocyte-associated antigen 4
SBRT	Stereotactic body radiotherapy
TMZ	Temozolomide
TCR	T-cell receptor
RANO	Response assessment in neuro-oncology
